# Increased water intake reduces long-term renal and cardiovascular disease progression in experimental polycystic kidney disease

**DOI:** 10.1371/journal.pone.0209186

**Published:** 2019-01-02

**Authors:** Priyanka S. Sagar, Jennifer Zhang, Magda Luciuk, Carly Mannix, Annette T. Y. Wong, Gopala K. Rangan

**Affiliations:** 1 Centre for Transplant and Renal Research, Westmead Institute for Medical Research, The University of Sydney, Sydney, NSW, Australia; 2 Department of Renal Medicine, Westmead Hospital, Western Sydney Local Health District, Sydney, NSW, Australia; Universita degli Studi di Bari Aldo Moro, ITALY

## Abstract

Polycystic kidney disease (PKD) is the most common inherited cause of kidney failure and currently has limited treatment options. Increasing water intake reduces renal cyst growth in the *pck* rat (a genetic ortholog of autosomal recessive PKD) but it is not clear if this beneficial effect is present in other models of PKD. In this study, we tested the hypothesis that high water intake (HWI) reduces the progression of cystic renal disease in Lewis polycystic kidney (LPK) rats (a genetic ortholog of human nephronophthisis-9). Groups of female and male LPK (n = 8–10 per group) and Lewis (n = 4 per group) rats received water *ad libitum* supplemented with or without 5% glucose [to simulate HWI or normal water intake (NWI) respectively] from postnatal weeks 3 to 16. Water intake increased ~1.3-fold in the LPK+HWI group compared to LPK+NWI rats between weeks 3 to 10 but the differences were not significant at later timepoints. In LPK rats, HWI reduced the increases in the kidney to body weight ratio by 54% at week 10 and by 42% at week 16 compared to NWI (both p<0.01). The reduction in kidney enlargement was accompanied by decreases in the percentage renal cyst area, percentage renal interstitial collagen and proteinuria (all p<0.05). At week 16, HWI reduced systolic blood pressure and the heart to body to weight ratio by 16% and 21% respectively in males LPK rats (both p<0.01). In conclusion, a modest increase in water intake during the early phase of disease was sufficient to attenuate renal cystic disease in LPK rats, with secondary benefits on hypertension and cardiovascular disease. These data provide further preclinical evidence that increased water intake is a potential intervention in cystic renal diseases.

## Introduction

Polycystic kidney disease (PKD) is the most common inherited cause of end-stage renal disease (ESRD) [1–3]. It is characterised by the formation and growth of numerous fluid-filled kidney cysts and cystic tubular dilatations, which cause progressive nephron obstruction, tubulointerstitial injury and renal impairment with secondary hypertension and cardiovascular disease [2]. Arginine vasopressin (AVP) is a neuro-hypophyseal hormone that regulates water homeostasis and is released in response to elevated serum osmolality [4]. In the *pck* rat (a genetic ortholog of autosomal recessive PKD), circulating AVP was critical for triggering the formation of renal cysts as well as contributing to their continued growth *via* activation of cyclic adenosine monophosphate (cAMP)-mediated transepithelial fluid secretion and cell proliferation [5–7]. Furthermore, pharmacological receptor antagonists of AVP, such as tolvaptan, reduce renal cyst growth and the decline in renal function in both experimental and human PKD, confirming the importance of AVP in renal cyst growth [3, 8].

Increasing the intake of water attenuates AVP release, and has been hypothesised to be an easily-accessible and safe therapeutic intervention to reduce renal cyst growth in PKD [2, 9, 10]. To date, there have been only two preclinical studies that have evaluated the efficacy of increased water intake in PKD [11, 12]. In this regard, Nagao *et al*. demonstrated that increased water intake (induced by adding 5% glucose to the drinking water) over 10 weeks reduced the progression of kidney enlargement and the percentage cyst area in *pck* rats [11]. Furthermore, Hopp *et al*. replicated these findings in the *pck* rat (using hydrated agarose gel and 5% glucose) but unexpectedly did not observe a protective effect in a hypomorphic model of autosomal dominant PKD (*Pkd1*^*RC/RC*^ mice) [12].

It is not known if the beneficial effects of high water intake attenuates the long-term progression of kidney enlargement in other models of PKD. The Lewis polycystic kidney (LPK) rat model is a hypertensive rat model of cystic renal disease due to a point mutation in *Nek8* and is genetically orthologous to human nephronophthisis (NPHP)-9 [13]. Interestingly, the renal phenotype of LPK rats varies from NPHP and is characterised by progressive nephromegaly (due to diffuse collecting duct ectasia from postnatal weeks 3 to 20), late-onset end-stage renal failure and hypertension, with similarities to autosomal recessive PKD [14, 15]. The LPK rat model provides a valuable preclinical tool to evaluate and understand the chronic efficacy of therapeutic interventions in PKD. In the present study, the hypothesis that an high water intake (HWI) reduces the progression of cystic renal disease in the LPK rat model, was investigated.

## Methods

### Animals

All animals were obtained from the breeding colony at Westmead Hospital and allowed food and water *ad libitum*. The study was approved by the Western Sydney Local Health District Animal Ethics Committee (Protocol number 4100) and conducted according to the Australian Code for the Care and Use of Animals for Scientific Purposes [16].

### Experimental design

**Fig 1:**
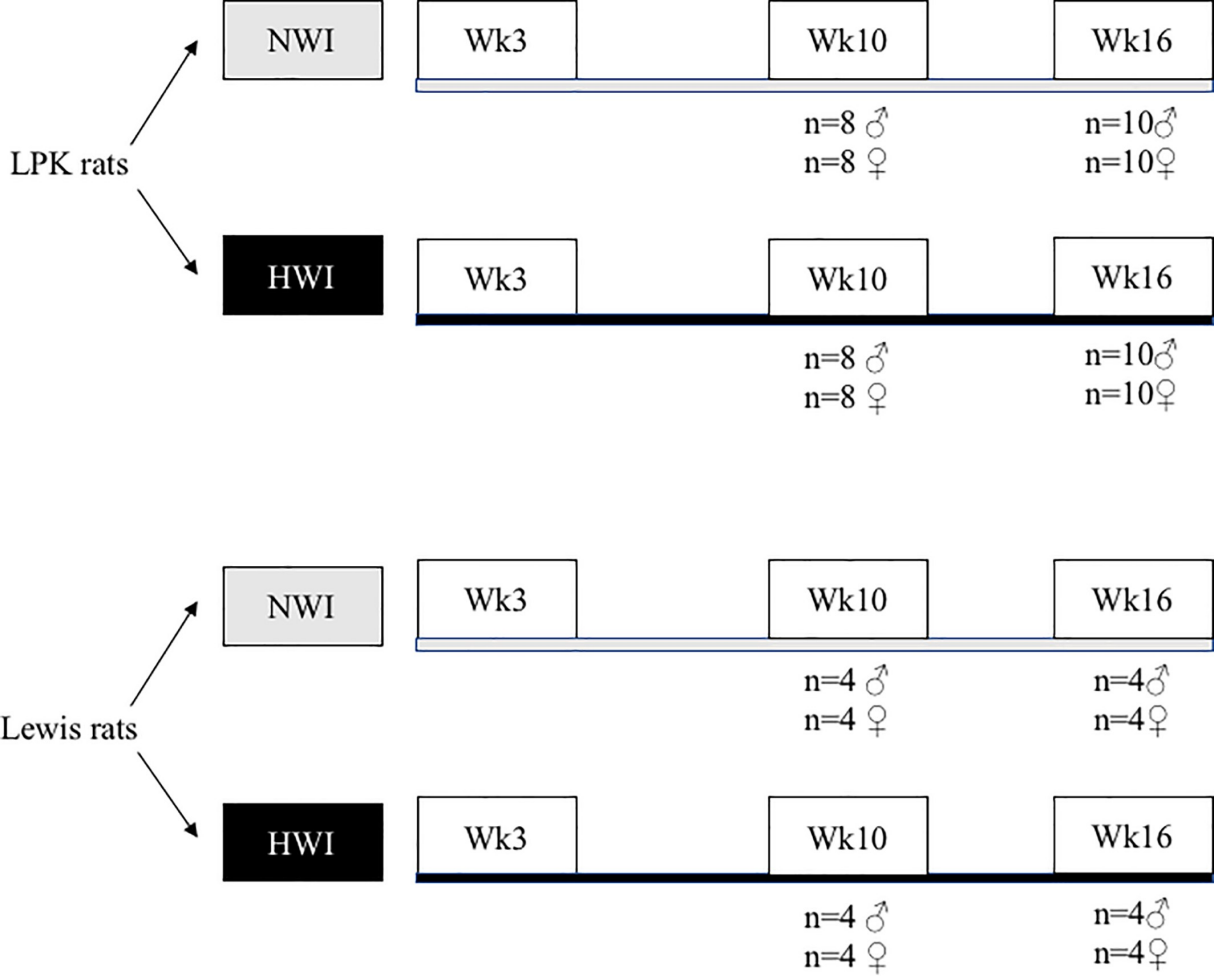
Experimental design. Female and male Lewis and LPK litter-mates were equally divided into two groups—HWI (tap water supplemented with 5% glucose) or NWI (normal water intake). The rats were sacrificed at either week 10 (LPK n = 33, Lewis n = 15) or week 16 (LPK n = 33, Lewis n = 17).

Following weaning, LPK and control Lewis littermates were equally divided into two groups that had free access to tap water either supplemented with or without 5% glucose [to simulate HWI or normal water intake (NWI) respectively] from postnatal week 3 until week 16 (Fig 1). Groups of rats were sacrificed at either week 10 or week 16 (LPK n = 66; Lewis n = 32). Both male and female animals were examined to determine the gender-specific effects of increased water intake. The sample size and time-points were based on the natural history of the LPK model, with the period between weeks 3 and 10 being characterised by rapid kidney growth (that is, early PKD), whereas the interval between weeks 10 to 16 marking the onset of progressive tubulointerstitial inflammation and fibrosis, hypertension and worsening renal function (that is, late PKD) [15].

The rats were placed in metabolic cages for 14 hours, at weeks 10 and 16, and sacrificed on the following day. At the time of sacrifice, rats were anaesthetised by an intraperitoneal injection of ketamine/xylazine, blood was collected by cardiac puncture, and both kidneys and heart were rapidly removed by surgical dissection as described in previous studies [15, 17].

The addition of 5% glucose to drinking water is a well-established model to increase water intake and is not known to have any detrimental effects [11, 12]. It been used in several previous studies to investigate the effects of HWI on the progression of PKD and CKD in small animal models [11, 12]. Moreover, Hopp *et al*. validated that tap water supplemented with 5% glucose was equivalent to providing food enriched with hydrated agarose gel on the efficacy on renal cyst growth in the *pck* rat [12].

### Measurement of water intake

Water intake was assessed by two methods during the study. In the first method, water intake was measured in each rat at two time-points (weeks 10 and 16) while they were *individually-housed* in metabolic cages (for 14 hours). In the second method, the consumption of water was measured continuously for entire the duration study while rats were *group-housed* in standard cages (n = 3–4 littermates) by measuring the weight of water bottles. In this method, water intake was recorded every morning (between 0700–0900 hrs) second daily (arbitrarily designated Day 0 and Day 2) and calculated by the following formula:

$$\mathrm{Water}\;\mathrm{intake}\;\mathrm{per}\;\mathrm{rat}/\mathrm{day}\;\left(\mathrm{mls}\right)=\frac{\left(\mathrm{Bottle}\;\mathrm{Weight}\;\mathrm{on}\;\mathrm{Day}\;0-\mathrm{Bottle}\;\mathrm{Weight}\;\mathrm{on}\;\mathrm{Day}\;2\right)}{\left(\mathrm{Number}\;\mathrm{of}\;\mathrm{Rats}\;\mathrm{in}\;\mathrm{Cage}\;X\;\mathrm{No}.\mathrm{of}\;\mathrm{Days}\;\mathrm{Since}\;\mathrm{Last}\;\mathrm{Measurement}\right)}$$

### Renal function and other biochemistry

The serum urea, creatinine, random glucose, and osmolality were measured from blood collected from all rats at the time of sacrifice, as previously described [15][17]. The urine volume, creatinine and osmolality were measured from samples collected while rats were placed in metabolic cages on the day prior to sacrifice. The creatinine clearance was corrected for body weight, and calculated as described previously [15, 17]. The serum sodium and albumin were measured in a subset of rats from each group. In addition, the osmolyte intake was estimated by multiplying the urine volume with urinary osmolality.

### Histology and quantitative image analysis

Coronal sections of kidney and heart were fixed in either methyl carnoy solution or 10% neutral buffered formalin, and embedded in paraffin. Tissue sections for histology or immunohistochemistry were cut at 4μm in thickness. Whole-slide imaging of stained slides was performed (magnification x 20) using a digital slide-scanner (Hamamatsu Nanozoomer, Hamamatsu Photonics, Shizuoka, Japan). To determine the renal cyst area, methyl carnoy-fixed samples were stained with Periodic-Acid Schiff (PAS) and the cystic (‘white’) areas was quantified by the area morphometric analysis using imaging analysis software (Aperio Imagescope, v12.3.2.8013 Leica Biosystems, Wetzlar, Germany). The proportion occupied interstitial fibrosis in renal and cardiac tissue was quantified by Sirius Red collagen staining. Renal inflammation was determined by interstitial monocyte and myofibroblast accumulation, using antibodies reactive against either CD68 (1:400, MCA341R; Serotec, Kidlington, U.K.) or α-smooth muscle actin (α-SMA) (1:1000, A2547; Sigma–Aldrich, St. Louis, MO, USA) respectively, as previously described [18]. All immunohistochemical stains were analysed in whole-slide images using a standardized algorithm to measure positive pixels in the Aperio Imagescope software. The percentage monocyte and myofibroblast infiltration, and interstitial collagen deposition indexes were calculated using the formula: $\left(\frac{Positive\;Pixels}{Total\;Pixels}\right)\times 100$. The surface area of myofibroblasts, monocyte or interstitial collagen deposition was calculated per mm^2^ by the formula: $\left(\frac{Positive\;Pixels\;x\;Total\;Section\;Area}{Total\;Pixels}\right).$

### Evaluation of blood pressure and cardiovascular disease

To assess the progression of cardiovascular disease in LPK rats, serial tail-arterial blood pressure measurements were performed in male Lewis (n = 8) and male LPK rats (n = 14) between weeks 14 to 16 of age as previously described [15, 17]. In addition, cardiac weight was measured in all rats at the time of sacrifice and corrected for body weight.

### Statistical analysis

The data was analysed using JMP statistical software and results presented as mean ± SD. A *P* value less than 0.05 was defined as statistically significant. The differences between LPK and Lewis groups were analysed by the Tukey-Kramer (HSD) or Wilcoxon each pair test.

## Results

### General effects of increased water intake

Both Lewis and LPK rats tolerated increased water intake (induced by 5% glucose in drinking water) with no apparent adverse effects, and no deaths occurred during the study. In general, as expected, male rats weighed significantly more than female rats in all groups (p<0.01, S1 Table). Moreover, the body weight of male Lewis rats was higher when compared to male LPK male rats, whereas no difference was observed between female Lewis and female LPK rats.

When comparing the NWI and HWI groups, there was no differences in body weight between the LPK and Lewis groups at the commencement of the intervention (at week 3). In addition, the growth of Lewis rats between week 3 and 10 or 16 were the same in both the HWI and NWI groups. In LPK male rats, there was no difference in body weight at week 10 between the HWI and NWI groups. However, at week 16, males in the LPK+HWI group had a higher body weight compared to the LPK+NWI group (p = 0.01, S1 Table), whereas there was no difference for female LPK rats. Also, as shown in Table 1, the serum glucose and albumin were the same between HWI and NWI group at both timepoints for both Lewis and LPK rats.

**Table 1:** Effects of increased water intake on body weight, kidney weight, kidney cyst area, and serum glucose and albumin.

	Lewis		LPK	
	NWI	HWI	NWI	HWI
Week 10	n = 8	n = 8	n = 17	n = 16
*Body weight at week 3 (g)*	32±3	31±3	31±6	32±5
*Body weight at week 10 (g)*	202±56	185±44	174±29	188±32
*Kidney*: *body weight (%)*	0.8±0.0	0.7±0.0	7.3±0.8*	3.4±0.7†
*Kidney cyst area (mm*^*2*^*)*	7.7±3.3	5.7±112.8	112.8±15.4*	59.8±15.0†
*Kidney section area (mm*^*2*^*)*	45.4±11.1	38.0±4.0	174.7±22.5*	111.5±20.0†
*Kidney cyst area*: *kidney section area (%)*	17.1±5.7	14.9±5.2	64.6±4.6*	53.4±5.3†
*Serum glucose (mmol/L)*	13±2.4	12.7±2.4	10.7±2.6	12.6±4.2
*Serum albumin (g/L)*	29±3	29±1	29±4	29±2
**Week 16**	**n = 9**	**n = 8**	**n = 17**	**n = 16**
*Body weight at week 3 (g)*	35±4	34±2	31±7	31±6
*Body weight at week 16 (g)*	241.1±66.8	250.4±70.8	211.5±49	257±63.5
*Kidney*: *body weight (%)*	0.7±0.1	0.7±0.0	7.8±1.0*	4.4±0.9†
*Kidney cyst area (mm*^*2*^*)*	9.0±4.9	8.7±3.5	143.4±29.4*	103.1±31.6†
*Kidney section area (mm*^*2*^*)*	46.4±11.9	46.7±10.4	210.2±40.6*	163.5±38.6†
*Kidney cyst area*: *kidney section area (%)*	18.6±5.5	18.0±4.0	68.2±4.6*	61.9±6.6§
*Serum glucose (mmol/L)*	12.1±3.2	13±2.2	10±2.3	10.6±1.9
*Serum albumin (g/L)*	30±1	33±1	31±3	31±3

NWI, normal water intake; HWI, high water intake; KW:BW% (mg), kidney weight to body weight ratio expressed as a percentage. Values represented as mean ± SD.

*p<0.001 versus age-matched NWI Lewis rat

†p<0.001 versus age-matched LPK NWI rat

‡p<0.05 versus age-matched NWI Lewis rat

§p<0.05 versus age-matched NWI LPK rat. Unless otherwise indicated differences between the groups are not statistically significant

### Changes in water consumption in the experimental groups

#### Water consumption while rats were housed individually in metabolic cages

As discussed in the Methods, water consumption was assessed in two ways: (i) while rats were housed individually in metabolic cases at two specific timepoints prior to sacrifice (i.e. weeks 10 and 16); and (ii) while rats were group-housed in standard cages (discussed below). The data for water intake is shown in Table 2. In general, LPK+NWI rats had a higher water intake than Lewis+NWI rats (~1.4-fold higher at week 10, p = 0.0043; and 1.7-fold more at week 16, p = 0.0368), most likely due to loss of urinary concentrating ability and increased thirst response in PKD-affected LPK rats compared to normal Lewis rats [19, 20]. When comparing the HWI and NWI groups, Lewis+HWI rats had a 1.8- and 3.7-fold increase in water intake compared to the Lewis+NWI at week 10 and 16 respectively (both p<0.0001; Table 2). Similarly, in the LPK+HWI group, water intake increased 1.7-fold compared to the LPK+NWI group at week 10 (p<0.0001), and at week 16 the magnitude of increase in water intake was ~1.2-fold (p = 0.0963). The estimated osmolyte intake was similar in all groups at week 10, but at week 16 it was reduced in LPK+NWI group compared to the Lewis+NWI group (Table 2).

**Table 2:** Changes in water intake (as determined while housed in metabolic cages) and urine osmolality, estimated osmolyte intake and serum sodium/urea.

	Lewis		LPK	
*Variables*	**NWI**	**HWI**	**NWI**	**HWI**
**Week 10**	**n = 8**	**n = 8**	**n = 16**	**n = 11**
*24 hr water intake (mls)*	29.5±7.7	54.7±13.8‡	41.1±7.6*	68.5±17.4†
*24 hr urine volume (mls)*	13.2±7.4	27.6±12.4	18.9±8.0	29.2±14.1§
*Urine osmolality (mmol/kg)*	987.8±606.5	451.1±184.2‡	588.4±158‡	434.2±82.5
*Estimated osmolyte intake (mmol/day)*	11.3+2.8	12.8+2.6	10.3+3.2	11.7+3.6
*Serum osmolality (mmol/kg)*	313.1±13.0	308.4±12.9	354.1±27.9‡	310.1±28.4†
*Serum sodium (mmol/L*^*)*^	144±2	142±3	147±4	136±11†
*Serum urea (mmol/L)*	6.5±1.5	5.6±0.8	20.2±5.8*	9.3±3.0†
**Week 16**	**n = 9**	**n = 6**	**n = 13**	**n = 12**
*24 hr water intake (mls)*	27.0±7.4	92.4±28.4	46.7±10.1*	64.4±18.1
*24 hr urine volume (mls)*	10.1±4.2	58.1±43.6*	20.2±8.2	29.2±9.7
*Urine osmolality (mmol/kg)*	1624.2±649.5	370.3±341.1*	481.4±179.7*	411.8±78
*Estimated osmolyte intake (mmol/day)*	14.2±2.1	12.4±3.6	9.3±3.6*	11.4±3.1
*Serum osmolality (mmol/kg)*	324.6±44	311±14.9	356.2±32.4	324.9±24.4†
*Serum sodium (mmol/L)*	145±7	146±4	146±5.0	141±8
*Serum urea (mmol/L)*	7.2±1.5	5.8±0.9	29.0±8.9*	16.7±7.4†

Values represented as mean ± SD.

*p<0.001 versus age-matched NWI Lewis rat

†p<0.001 versus age-matched LPK NWI

‡p<0.05 versus age-matched NWI Lewis rat

§p<0.05 versus age-matched NWI LPK rat.

#### Water consumption while rats were housed in groups in standard cages

**Fig 2:**
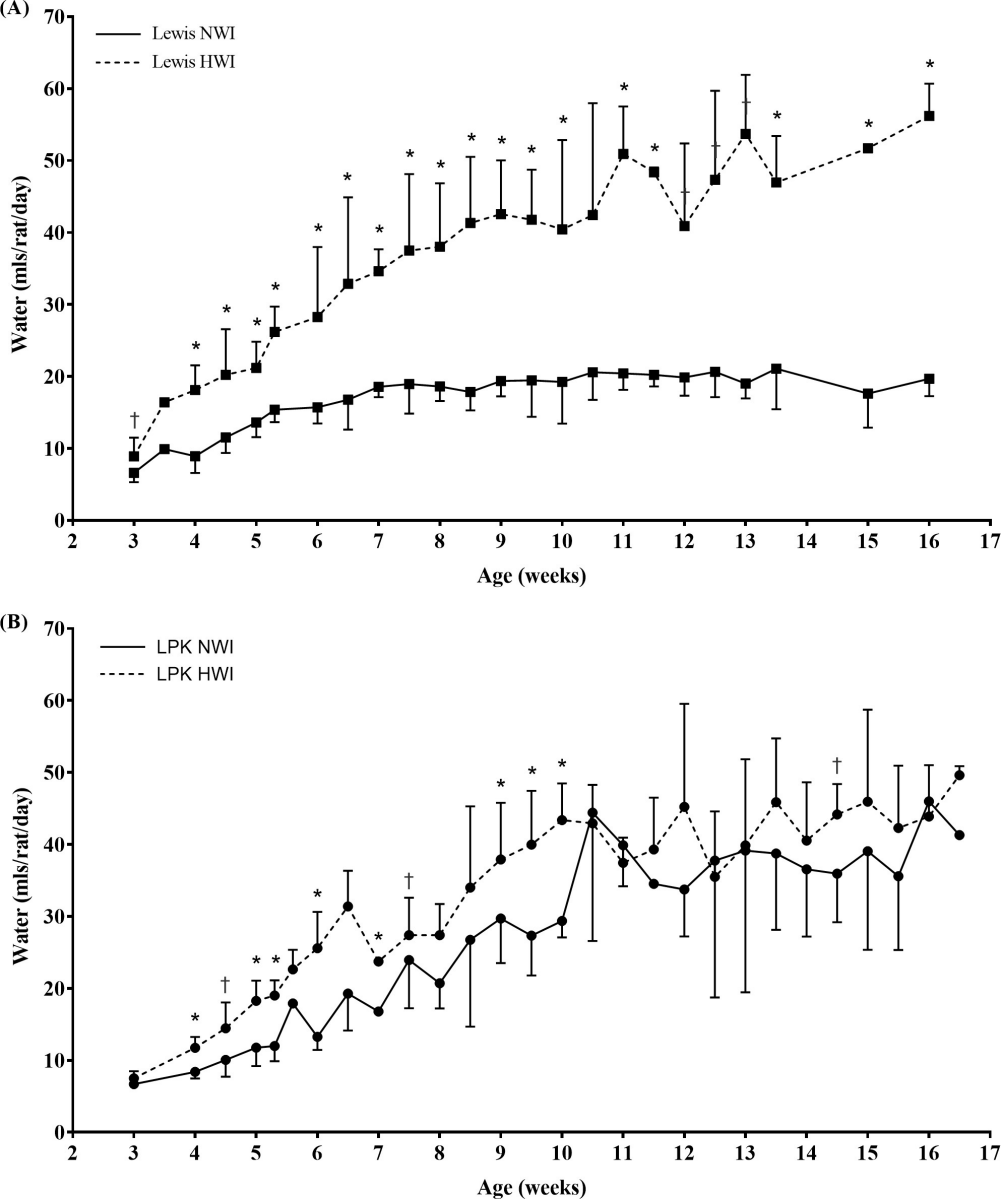
Water intake while rats were group housed in standard cages. (A) Water intake in the Lewis rat groups. Water intake in the Lewis+HWI rats was significantly increased water intake at all timepoints during the study period compared to the NWI group. *p<0.001 and †p<0.05 versus age-matched NWI Lewis rat. (B) Water intake in the LPK rat groups. Water intake in the LPK+HWI group was increased only up to week 10, after which it was similar to LPK+NWI rats. *p<0.001 and †p<0.05 versus the age-matched NWI group. Please see Methods for details on calculation.

To provide serial measurements of fluid intake during the study, water consumption was also assessed continuously while rats were group-housed in standard cages, as shown in Fig 2. Overall, for both Lewis and LPK rats, water intake in the HWI groups (n = 48) was at least 1.3-fold higher than the NWI groups. For Lewis rats, the increase in water intake in the HWI group was statistically significant at all timepoints throughout the study period (Fig 2A). However, for the LPK+HWI group water consumption was higher compared to the LPK+NWI until week 10 (p<0.0001, Fig 2B). After week 10, the water intake of the LPK+HWI group was not consistently higher than LPK+NWI rats (p = 0.9888).

### Effect of increased water intake on urine volume and biochemistry

#### Urine volume and urine osmolality

The results for urine volume and osmolality while rats were housed individually in the metabolic cages are shown in Table 2. In general, the urine volume tended to be lower than the volume of water consumed possibly in part due to behavioural factors and/or urine evaporation in the metabolic cage [21, 22]. In Lewis rats, there was a trend for an increase in urine volume in the HWI group at week 10 (p = 0.0728) and this statistically significant at week 16 (5.8-fold; p<0.0001) when compared to the NWI group. In LPK rats, the urine volume increased 1.5-fold in HWI group (p = 0.0484) at week 10 but was not statistically different at week 16 (p = 0.5829) compared to NWI group.

The changes in urine osmolality in the experimental groups are shown in Table 2, and were consistent with the effects of increased water intake but also highlighted the effect of renal disease progression in PKD. Comparing the NWI groups, the urine osmolality was higher in Lewis rats compared to LPK rats at both week 10 and 16 (p<0.0001) most likely due to the loss of urinary concentrating ability in PKD [19, 20]. In the Lewis rat groups, there was a significant decrease in urine osmolality with HWI compared to NWI at both timepoints (p<0.0001),. In contrast, in LPK rats, there was no difference between the LPK+HWI and LPK+NWI groups at either week 10 (p = 0.5503) or week 16 (p = 0.9256), indicating that it is a less sensitive biomarker to gauge the serial effects of changes in fluid intake in PKD, as suggested in previous studies [23].

#### Serum osmolality, sodium and urea

As shown in Table 2, the serum osmolality and urea were higher in LPK+NWI rats compared to Lewis+NWI rats, possibly secondary to increased urinary loss of solutes in PKD [20]. In Lewis rats, serum osmolality, urea and sodium were not altered in the HWI groups compared to the NWI rats (Table 2). In contrast, in LPK+HWI rats the serum osmolality and urea were significantly lower compared to LPK+NWI rats at both time points (p<0.0001). Interestingly at week 10, this decrease was also associated with a reduction in the serum sodium in the LPK+HWI group compared to LPK+NWI (p = 0.0071), but statistical significance was not present at week 16 (p = 0.4692).

### Effect of increased water intake on kidney enlargement and renal cystic disease

#### Kidney enlargement

The results for kidney enlargement (as determined by the kidney to body weight ratio) are shown in Table 1. The kidney size was increased by 10- and 11-fold in LPK+NWI rats compared to the Lewis+NWI group at week 10 and 16 respectively (p<0.0001 for both). In Lewis rats, increased water intake did not alter kidney size. In contrast, in the LPK+HWI groups there was a 54% and 43% reduction in the progression of kidney enlargement at week 10 and 16 respectively compared to the LPK+NWI group (p<0.0001).

#### Percentage kidney cyst area

**Fig 3:**
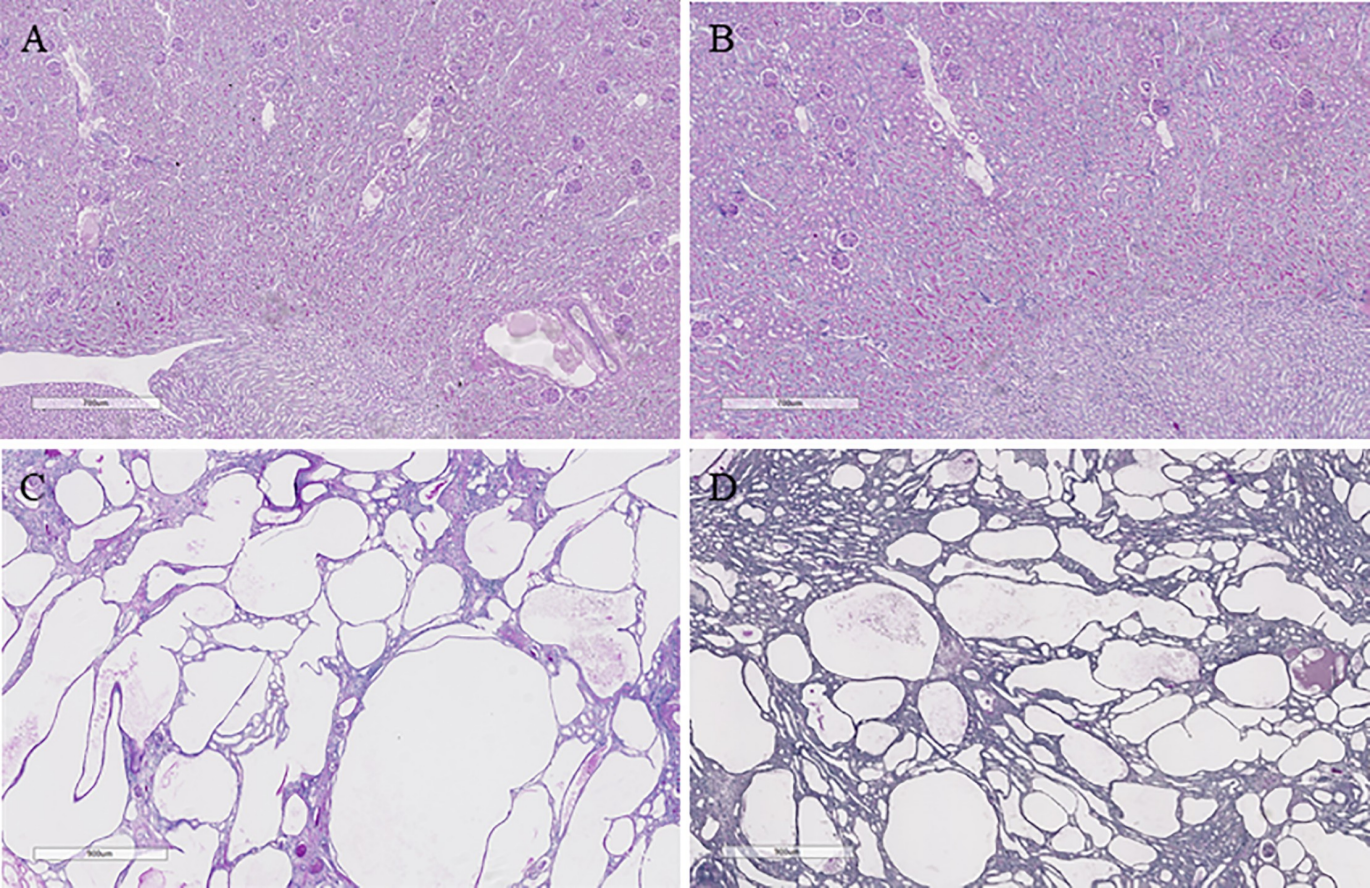
Representative photomicrographs of periodic acid Schiff-stained sections of kidney showing effects on cyst growth at week 10 in Lewis+NWI (A), Lewis+HWI (B), LPK+NWI (C) and LPK+HWI (D). There was no change in renal structure in the Lewis rats with HWI, whereas in LPK rats there was a marked attenuation in the renal cystic tubular dilation (D vs. C).

HWI attenuated the progression of renal cyst area in LPK rats, as determined by histological analysis. The percentage cyst area was reduced by 47% and 28% at week 10 and 16 respectively in LPK+HWI rats compared to the LPK+NWI group (both p<0.0001), with an overall decrease in cystic index percentage (cyst area: renal section area) by 17% and 9%, at week 10 and 16 (both p<0.0001) in the LPK+HWI compared to LPK+NWI (Table 1 and Fig 3).

#### Renal interstitial myofibroblast and monocyte accumulation

Renal interstitial myofibroblast and monocyte accumulation were examined by immunohistochemistry for α-SMA and CD68 respectively. As shown in Table 3, myofibroblast infiltration was increased in the LPK+NWI group (with 12% of total section area positively stained for myofibroblasts) compared with Lewis+NWI (where 3% of the total section area positively stained for myofibroblasts), at both timepoints (p = 0.0152). However, HWI did not alter the progression of myofibroblast accumulation in either LPK or Lewis rats, at either timepoint (p = 1.0000). Representative photomicrographs of α-SMA in LPK and Lewis groups are shown in S1 Fig. Similarly, interstitial monocyte (CD68+) infiltration was increased in the LPK+NWI groups compared to the Lewis+NWI rats (2.1-fold increase in positively stained monocyte area in LPK+NWI at week 10 (p = 0.0208) and 2.2-fold at week 16 (p = 0.0036)). However, HWI did not affect monocyte infiltration in either Lewis or LPK HWI groups in comparison to NWI groups. These results were unchanged when analysed according to gender (S2 Table).

**Table 3:** Effect of increased water intake on renal myofibroblast and monocyte infiltration and interstitial collagen deposition.

	Lewis		LPK	
*Variables*	NWI	HWI	NWI	HWI
**Week 10**	**n = 7**	**n = 8**	**n = 17**	**n = 15**
*Renal section area (mm*^*2*^*)*	45.8±11.3	39.8±4.5	173.1±24.5*	110.5±18.5†
*Myofibroblast infiltration (mm*^*2*^*)*	1.24±1.1	1.17±0.5	9.76±5.4‡	6.60±3.5
*Myofibroblast index (%)*	3.2±3.0	3.3±1.5	12.3±7.1‡	10.4±4.5
*Monocyte infiltration (mm*^*2*^*)*	0.33±0.2	0.33±0.3	0.69±0.5‡	0.62±0.3
*Monocyte index (%)*	1.0±0.4	1.1±0.9	1.2±0.7	1.3±0.6
*Interstitial collagen deposition (mm*^*2*^*)*	3.6±2	1.6±1.3	27.5±17.8	17.1±7.4
*Interstitial collagen deposition index (%)*	7.6±3.1	4.3±3.7	16.2±11.4	15.4±6.0
**Week 16**	**n = 7**	**n = 8**	**n = 17**	**n = 15**
*Renal section area (mm*^*2*^*)*	45.9±11.6	46.6±10.1	214.5±41.5*	158.6±39.2†
*Myofibroblast infiltration (mm*^*2*^*)*	1.29±0.6	1.08±0.6	11.2±6.6‡	10.2±5.1
*Myofibroblast index (%)*	3.0±1.2	2.4±1.3	12.4±7.5‡	12.6±6.2
*Monocyte infiltration (mm*^*2*^*)*	0.26±0.2	0.33±0.4	0.56±0.3‡	0.45±0.1
*Monocyte index (%)*	0.7±0.5	0.9±1.1	0.8±0.5	0.8±0.3
*Interstitial collagen deposition (mm*^*2*^*)*	3.9±2.5	2.8±2.0	51.5±33.9*	25.1±10.7§
*Interstitial collagen deposition index (%)*	8.7±5.0	5.9±4.1	25.0±17.6‡	16.6±7.9

*p<0.001 versus age-matched NWI Lewis rat

†p<0.001 versus age-matched LPK NWI

‡p<0.05 versus age-matched NWI Lewis rat

§p<0.05 versus age-matched NWI LPK rat

#### Renal fibrosis

**Fig 4:**
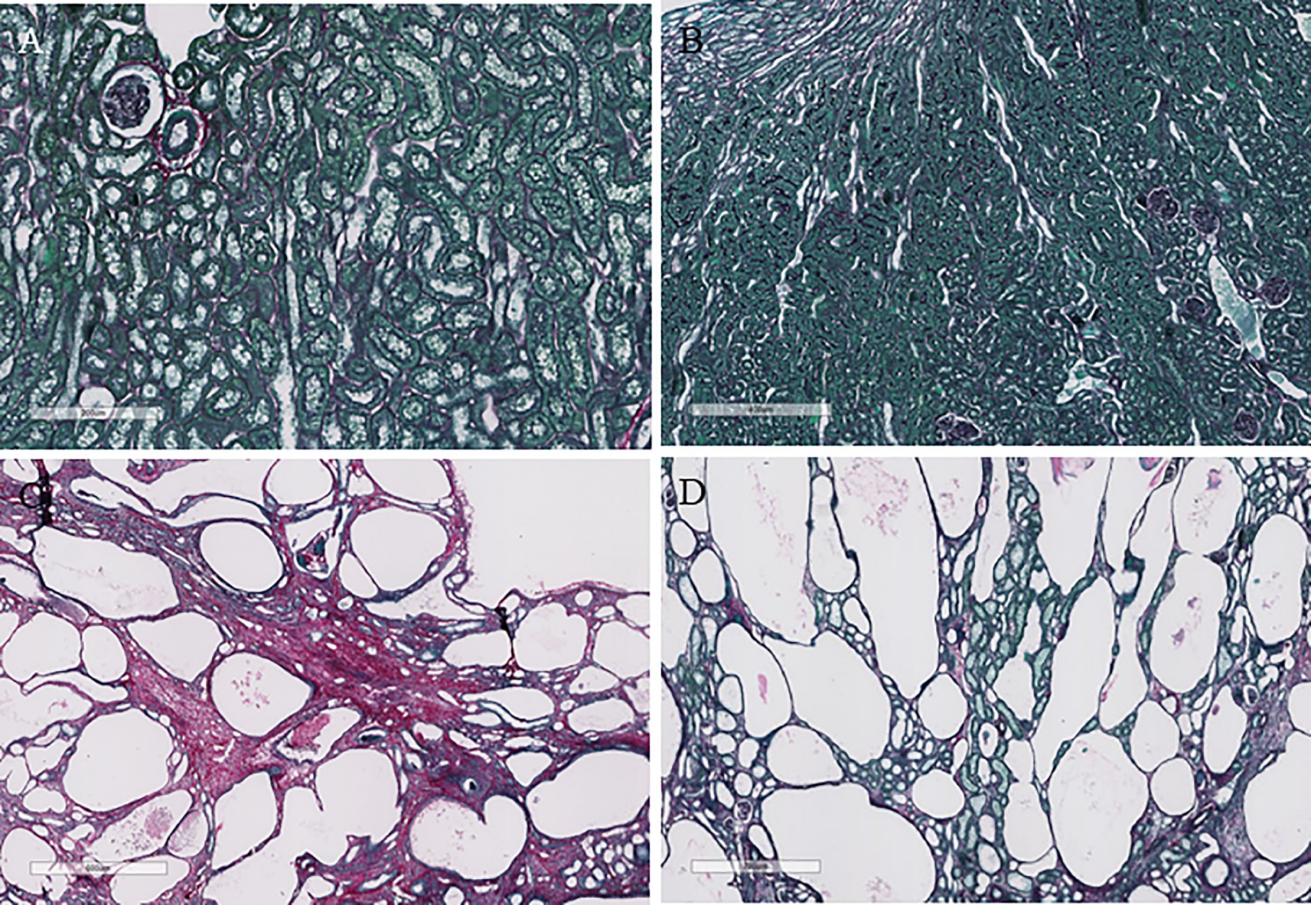
Representative photomicrographs of Sirius red stained sections of kidney tissue showing interstitial collagen deposition at week 20 in Lewis+NWI (A), Lewis+HWI (B), LPK+NWI (C) and LPK+HWI (D). There was no change in renal fibrosis in the Lewis rats with increased water intake, whereas in LPK rats (D) there was a marked attenuation in interstitial collagen deposition in comparison to LPK+NWI (C).

Renal fibrosis as determined by Sirius red staining for collagen deposition, was increased in LPK+NWI rats at week 16 (p<0.0001; Table 3 and Fig 4) but not at week 10 (p = 0.1023) when compared to Lewis+NWI. In LPK rats, interstitial collagen deposition was not altered by HWI at week 10, but at week 16, it was significantly reduced (by 50%; p = 0.0043) when compared to NWI (Table 4 and Fig 4). There were no changes between Lewis NWI and HWI groups (Table 4 and Fig 4).

**Table 4:** Effect of increased water intake on renal function.

	Lewis		LPK	
*Variables*	NWI	HWI	NWI	HWI
**Week 10**	**n = 8**	**n = 8**	**n = 17**	**n = 16**
*Serum creatinine (μmol/L)*	26±6	26±4	47±23	49±13
*CrCl/BW (ml/min/g)*	6.4±1.9	7.4±1.7	2.8±0.9*	3.0±1.1
*Urine PCR (mg/mmol Cr)*	39.8±29.9	116.8±88.9	297.7±499.0	334.2±261.4
**Week 16**	**n = 8**	**n = 8**	**n = 17**	**n = 16**
*Serum creatinine (μmol/L)*	33±10	33±9	93±42*	71±31
*CrCl/BW (ml/min/g)*	5.7±1.4	5.4±1.6	1.7±1.4*	2.0±1.4
*Urine PCR (mg/mmol Cr)*	23.1±15.3	149.4±101.1	1171.2±1387.8‡	346.6±370.4§

Urine PCR; urine protein creatinine ratio; CrCl/BW, creatinine clearance corrected for body weight. Values represented as mean ± SD.

*p<0.001 versus age-matched NWI Lewis rat

†p<0.001 versus age-matched LPK NWI

‡p<0.05 versus age-matched NWI Lewis rat

§p<0.05 versus age-matched NWI LPK rat

### Effects of increased water intake on renal function and proteinuria

#### Serum creatinine and creatinine clearance

LPK rats developed significant and progressive renal dysfunction over time (as determined by elevation in the serum creatinine and reduction in the endogenous creatinine clearance) compared to Lewis rats (Table 4). HWI did not affect renal function in Lewis rats. Similarly, in LPK rats the decline in renal function was not affected by HWI (Table 4 and S3 Table). Both LPK groups developed significantly elevated serum creatinine compared to Lewis rats at week 16 (p<0.0001). There was no difference in serum creatinine or calculated creatinine clearance corrected for body weight between HWI and NWI in either Lewis or LPK groups at both time points (Table 4) or when sub-analysed by gender (S3 Table) (p>0.2 in all cases).

#### Proteinuria

As shown in Table 4, there was a significant increase in proteinuria in the LPK+NWI rats compared to the Lewis+NWI group (p = 0.0263). In LPK rats, HWI reduced proteinuria by 70% (p = 0.0117) at the week 16 but not at week 10 (Table 4). There was no difference in proteinuria between the Lewis+HWI and Lewis+NWI groups at either timepoint (Table 4).

### Effects of high water intake on cardiovascular disease

#### Systolic blood pressure

**Fig 5:**
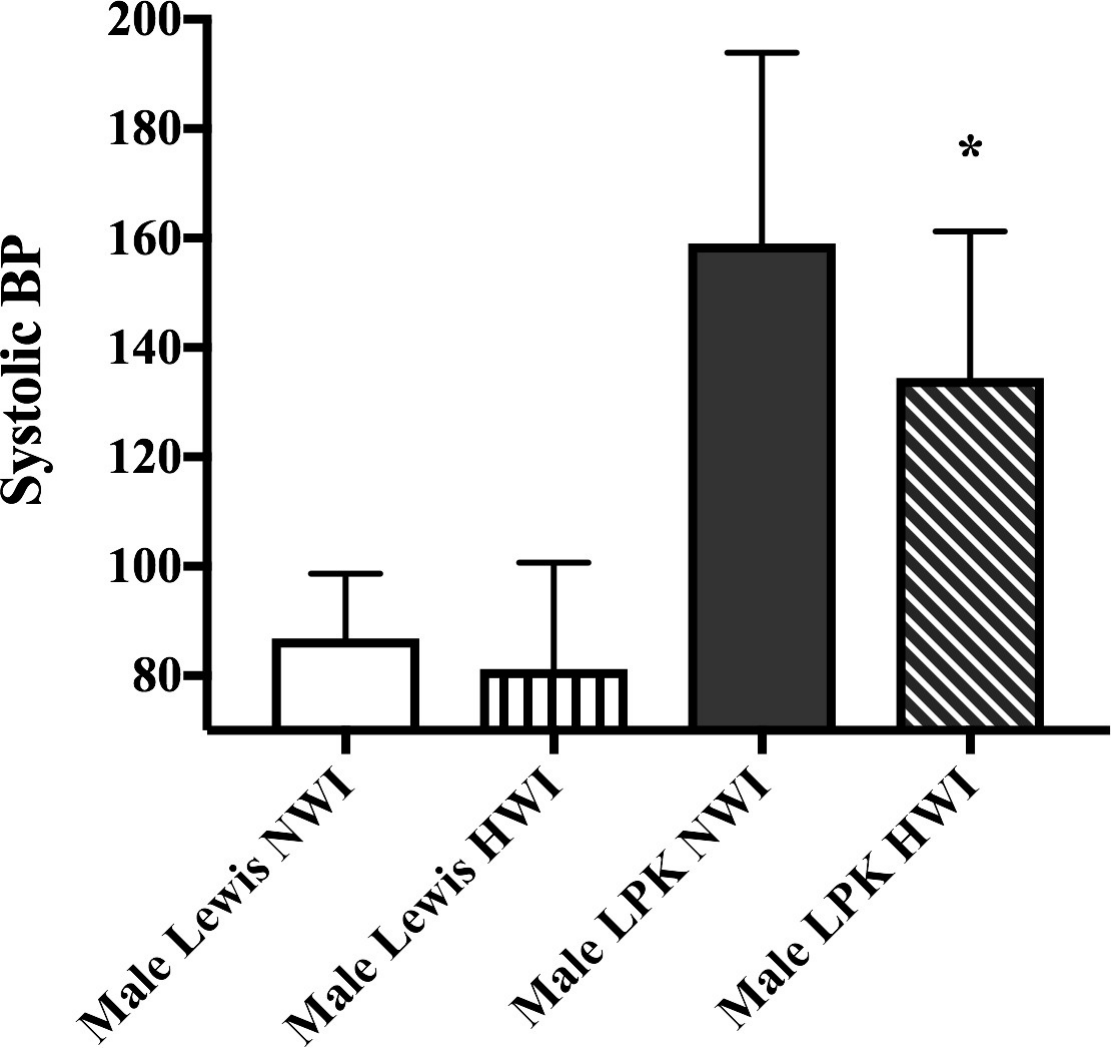
Effect of increased water intake on systolic blood pressure at week 16 in male Lewis and LPK rats. The data is expressed as mean ± SD (n = 4 for Lewis+NWI; n = 4 for Lewis+HWI; n = 8 for LPK+NWI, n = 10 for LPK+HWI); *p<0.01 when compared to Male LPK NWI group.

The effects of HWI on systolic blood pressure in male animals are shown in Fig 5. The systolic blood pressure was higher in LPK+NWI rats compared to Lewis+NWI group at week 16 (Lewis+NWI: 86.8±5.9; LPK+NWI: 159±14 mmHg; p<0.0001). In LPK rats at week 16, HWI reduced the systolic blood pressure by 15% (LPK+HWI: 134±9 mmHg) when compared to LPK+NWI (p<0.0022). There was no difference between Lewis+NWI and Lewis+HWI groups (Fig 5).

#### Cardiac enlargement and fibrosis

In LPK rats, cardiac enlargement is most likely due to the secondary effects of hypertension [14]. As shown in Table 5, there was a significant increase in cardiac enlargement (as determined by the heart weight to body weight ratio, HW:BW) in LPK+NWI group compared to Lewis+NWI rats at both week 10 and 16. Consistent with effects on systolic blood pressure, the LPK+HWI group had significantly reduced HW:BW ratio (by 21%, p<0.0082) compared with LPK+NWI at week 16. In contrast, increased water intake did not affect the HW:BW ratio in Lewis rats (Table 5).

**Table 5:** Effect of increased water intake on cardiac enlargement.

	Males				Females			
	Lewis		LPK		Lewis		LPK	
*Variables*	NWI	HWI	NWI	HWI	NWI	HWI	NWI	HWI
**Week 10**	**n = 4**	**n = 4**	**n = 7**	**n = 8**	**n = 3**	**n = 4**	**n = 9**	**n = 8**
*Cardiac weight (g)*	0.77±0.06	0.68±0.04	1.09±0.07*	1.14±0.08	0.48±0.08	0.51±0.03	0.86±0.07*	0.84±0.08
*HW*:*BW (%)*	0.31±0.03	0.3±0.001	0.55±0.07*	0.53±0.06	0.34±.03	0.35±0.02	0.57±0.06*	0.52±0.05
**Week 16**	**n = 4**	**n = 4**	**n = 8**	**n = 10**	**n = 5**	**n = 4**	**n = 9**	**n = 6**
*Cardiac weight (g)*	0.85±0.13	0.88±0.05	1.34±0.16*	1.34±0.12	0.64±0.07	0.57±0.13	0.97±0.13*	1.04±0.09
*HW*:*BW (%)*	0.28±0.02	0.28±0.02	0.57±0.10*	0.45±0.06§	0.34±0.02	0.30± 0.07	0.53±0.06*	0.57±0.06

HW:BW%: heart weight to body weight ratio expressed as a percentage. mean ± SD.

*p<0.001 versus age-matched NWI Lewis rat

†p<0.001 versus age-matched LPK NWI

‡p<0.05 versus age-matched NWI Lewis rat

§p<0.05 versus age-matched NWI LPK rat.

A subset of cardiac tissue from each rat group was also tested for collagen deposition with Sirius red staining, and there was no difference in fibrosis between NWI and HWI in both Lewis and LPK rats (S2 Fig).

## Discussion

PKD is an insidious and life-long disease with limited treatment options. The current study provides several new insights regarding the effects of increasing water intake in PKD: (i) an increase in water intake markedly attenuated long-term the progression of kidney enlargement, renal cyst growth and renal fibrosis in LPK rats, when initiated during the early stages of renal disease. It is noteworthy that while cystic renal disease was slowed it was not completely abrogated and the decline in renal dysfunction was not altered; (ii) remarkably, only a modest increase in water intake (between 1.4 to 1.7-fold increase above the control group) was sufficient to demonstrate a sustained benefit on kidney enlargement LPK rats; and (iii) finally, increased water intake also reduced systolic blood pressure and cardiac enlargement during the later stages of disease. These data extend previous results obtained in the *pck* rat and support the hypothesis that increased water intake reduces the progression of renal cyst growth in experimental PKD [11, 12].

AVP is a critical driver of postnatal kidney cyst growth, and the suppression of its endogenous production is considered a potential therapeutic intervention in PKD [5, 24]. The release of AVP from the pituitary gland is stimulated by an increase in the serum osmolality sensed by specific osmoreceptors [25]. As expected AVP levels have a close proportional relationship to serum osmolality in both humans and Lewis rats, that is reliable even if renal function is impaired. [4, 10]. This is not the case for other surrogate markers of AVP such as copeptin, which accumulates in renal failure [26]. Pharmacological receptor antagonists of AVP consistently reduce renal cyst growth and renal function decline in preclinical models and humans, but off-target effects (massive, rapid-onset and persistent aquaresis and increased risk for liver function derangement) are barriers for universal application in all patients with PKD [3, 27]. Thus, decreasing serum osmolality and subsequent release of endogenous AVP through increased water intake has been considered as an alternative therapy that is simple, easily accessible, and likely to have fewer adverse effects [24].

The efficacy of increased water intake on reducing kidney enlargement in LPK rats in this study is especially interesting because the efficacy is similar to that previously obtained with sirolimus (a very potent anti-proliferative agent), and thus highlights the importance of AVP in promoting cyst growth in PKD [28]. Two previous experimental studies have shown that increasing water intake in the *pck* rat was associated with a reduction in the progression of renal cystic disease [11, 12]. Nagao *et al*. measured the downstream effects of AVP and found the increased water intake reduced the expression of renal AVP V_2_ receptors, B-raf and ERK cellular proliferation pathways, and PCNA-positive renal cells likely driving the beneficial effects observed [11]. Similarly, Hopp *et al*. found that increased water intake downregulated ERK phosphorylation and renal cAMP production [12]. In the current study, there was a sustained reduction in serum osmolality (a surrogate marker for AVP) in the LPK+HWI group compared to the LPK+NWI rats at both the early and late stages of disease.

A surprising and unexpected finding of the present study was the relatively modest increase in daily water intake (1.4- to 1.9-fold increase) required to gain the beneficial effect, and this was lower than that observed in previous studies using the *pck* rat [11, 12]. The reasons for this discrepancy are not entirely clear but could be due to differences between physiological changes that occur in the animal models (*pck* rat vs. LPK rat) and methods of water intake measurements. For instance, Nagao *et al*. measured water intake only once during the study period in a metabolic cage at week 13.5 of age [11]. In contrast, Hopp *et al*. estimated water intake extrapolated from urine volume measured in metabolic cages at weeks 6, 8, and 10 of age [12], which may be influenced by urine concentrating capacity [19, 20].

In the present study, water intake was measured using two separate methods: (i) while housed individually in metabolic cages which we considered to be the gold-standard because it allows data measurement from each specific rat; and (ii) while rats were in standard housing cages which was the usual environment of the animals, and allowed continuous monitoring of fluid intake. While the pattern of consumption between the groups was the same between the housing and metabolic cages, the absolute volume of water consumed was increased in the metabolic cage compared to the housing cages (Table 2 and Fig 2). This was possibly due to a change in usual behaviour in the rats while in the individual metabolic cages, compared to the housing cages [22]. The difference in the volume of water consumed between the two environments may also contribute to the much higher targets of 3 to 4-fold increases in water intake reported in previous studies in comparison to this study. Additionally, there may be differences between the sensitivity of endogenous AVP to ingested water between the LPK and *pck* model that contributed to a lower water intake requirement to reduce serum osmolality and thirst drive. Further studies should be undertaken to determine if there is an optimal dose and duration of increased fluid intake to attenuate renal cyst growth in other animal models and also test this hypothesis in humans.

Although a detailed analysis of fluid intake was undertaken, a limitation of this study is that food intake was not measured. The assessment food intake is relevant given recent evidence showing that calorie restriction reduces renal cyst growth and fibrosis, and also that glucose metabolism is altered in renal cysts in PKD [29, 30]. That being said, the results of this study (including data for body weight, osmolality, serum glucose and albumin) do not suggest that differences in caloric intake or a metabolic effect of 5% glucose are likely to confound the observed effects on kidney enlargement. This is because there were no changes in body weight between the HWI and NWI groups. In relation to this, sub-analysis by gender showed that in male rats, body weight was reduced in the LPK+NWI group compared to LPK+HWI at week 16 (S1 Table). Two possible explanation are as follows: (i) food intake was reduced secondary to renal failure-induced anorexia, as serum urea was elevated in the LPK+NWI group compared to LPK+HWI rats (Table 2 and S3 Table). The absence of changes in serum albumin between these two groups does not necessarily argue against this hypothesis, given that is an insensitive marker of nutrition; (ii) net caloric intake was higher in the LPK+HWI groups given the addition of 5% glucose to drinking water, leading to body weight gain. Moreover, regardless of the mechanism, the attenuating effects of 5% glucose on kidney enlargement were observed in all LPK groups, regardless of gender and in the absence of an increase in body weight. These data, in combination with the results showing that there was a sustained decrease in serum osmolality in the LPK+HWI rat groups (Table 2), suggest that the findings are consistent with differences in hydration status between the groups [19].

Despite an improvement in renal cystic disease in the LPK+HWI rats the decline in renal function was not affected. Similarly, Hopp *et al*. did not demonstrate a change in serum creatinine with 5% glucose, and Nagao *et al*. reported a change in serum urea which may reflect hydration, but creatinine levels were not measured [11, 12]. The absence of a benefit of increased water intake on renal function in LPK rats is consistent with previous studies from our laboratory (using other therapeutic agents such as sirolimus), and likely reflects the persistent changes in abnormal cystic microarchitecture that continued in increased water intake groups despite reduction in cyst size [15, 31]. In addition, the sustained activation of non-AVP related growth factors may also contribute to renal impairment and sustained renal inflammation as demonstrated by the persistent α-SMA and ED-1+ cell accumulation seen in LPK+HWI rats, which was not suppressed by increased water intake [32, 33].

An important observation in the current study is that increased water intake reduced hypertension in male LPK rats. The mechanism of this effect could be related to the two main sites of action of AVP; at vascular smooth muscle where it causes vasoconstriction, and at the renal distal tubule where it increases blood volume, cardiac output and arterial pressure through activation of the protein kinase B (Akt)/mammalian target of rapamycin (mTOR) pathway [28, 34]. In cystic renal diseases, AVP-mediated stimulation at these sites result in hypertension and associated cardiovascular disease (such as concentric cardiac hypertrophy and diastolic impairment) [28, 34]. The beneficial effects observed in this study could be due to inhibition of these pathways. This finding is in keeping with clinical trials which showed that the use of AVP antagonists improved of hypertension and heart failure [35]. Given the cardiovascular disease burden in PKD, these results provide an important off-target protective benefit of increased water intake. The effect of increased water intake on blood pressure was also mirrored by a reduction in the heart to body weight ratio. While these data are supportive of the reduction in hypertension, a caveat that must be considered is that the absolute heart weight was similar in both groups, and therefore the differences in the ratio in male rats could simply be due to the body weight (S1 Table).

The strengths of the current study include the long duration of the study (which allowed sampling of the effects of hydration at an intermediate and late stage of PKD), the study of cardiovascular disease biomarkers (hypertension and the HW:BW ratio), the high frequency of water intake measures to ensure accurate recording of increased water consumption, the detailed renal histological analysis and finally the inclusion of both male and female animals which allowed us to confirm that there was no gender-bias on the protective effects of increased water intake. There were also several limitations; (i) while plasma osmolality reliably correlates with a reduction in AVP, no downstream markers of AVP measured; (ii) as discussed earlier, caloric intake and food consumption were not determined, but as mentioned above, the effects of this on the current study is unlikely to be significant; (iii) other methods to induce increase water intake (such as agarose gel) were not examined, and (iv) finally, the effects of increased water intake during the later phase of PKD (that is, between week 10 and 16) were not investigated.

In conclusion, the results of this study show that increased water intake reduces the progression of renal disease in the LPK rat model of PKD. Uniquely, our study shows that, an early and relatively modest increase in water intake is sufficient for long-term beneficial effects in LPK rat, and that the intervention also reduces systolic blood pressure and cardiovascular enlargement. Future preclinical and clinical studies should determine: (i) whether there is optimal amount and duration of increased water intake for slowing renal cystic renal disease; (ii) the comparative efficacy of increased water intake compared to pharmacological receptor blockade of AVP; (iii) the additive effects (if any) of increased water intake with other proven therapeutic interventions, and (iv) finally the mechanisms underlying the lowering of systolic blood pressure with increased water intake in PKD. Hitherto, few preclinical studies have investigated the effects of increased water intake, but the results of the current paper suggest that this line of investigation could be of immense value to the PKD community and other stakeholders.

## Supporting information

S1 Table Gender and effect of increased water intake on body weight, kidney weight, and cyst size at week 10. (DOCX)

S2 Table Gender and effect of increased water intake on renal inflammation and myofibroblast infiltration at week 10. (DOCX)

S3 Table Gender and effect of increased water intake on renal function, serum sodium and albumin. (DOCX)

S1 Fig Representative photomicrographs of alpha-smooth muscle actin stained sections of renal tissue at week 10. (A) Lewis NWI, (B) Lewis HWI, (C) LPK NWI, (D) Lewis HWI. Please see methods section for further details on staining protocol.
(TIF)

S2 Fig Representative photomicrographs of sirius red stained sections of cardiac tissue at week 16. (A) Lewis NWI, (B) Lewis HWI, (C) LPK NWI, (D) Lewis HWI. Red-stained areas are consistent with the deposition of collagen. Please see methods section for further details on staining protocol.
(TIF)
