# Supplementary material for: Increased water intake reduces long-term renal and cardiovascular disease progression in experimental polycystic kidney disease
Source: PLoS One. 2019 Jan 2;14(1):e0209186. doi: 10.1371/journal.pone.0209186 (PMC6314616; doi:10.1371/journal.pone.0209186)
Supplement: S1 Table — (DOCX) [file pone.0209186.s001.docx]

**Supplementary Data**

**S1 Table: Gender and effect of increased water intake on body weight, kidney weight, and cyst size at week 10**

|  | **Males** | | | | **Females** | | | |
| --- | --- | --- | --- | --- | --- | --- | --- | --- |
|  | **Lewis** | | **LPK** | | **Lewis** | | **LPK** | |
| *Variables* | **NWI** | **HWI** | **NWI** | **HWI** | **NWI** | **HWI** | **NWI** | **HWI** |
| **Week 10** | **n = 4** | **n = 4** | **n = 8** | **n = 8** | **n = 3** | **n = 4** | **n = 9** | **n = 8** |
| *Body weight (g)* | 246±16 | 225±17 | 200±18* | 216±19 | 143±11 | 145±6 | 150±10 | 161±7 |
| *Kidney weight (g)* | 1.9±0.1 | 1.6±0.2 | 14.3±1.5* | 7.4±1.3† | 1.2±0.1 | 1.1±0.0 | 11.1±1.1* | 5.2±0.8† |
| *Kidney: body weight (%)* | 0.79±0.01 | 0.73±0.03 | 7.18±0.86* | 3.49±0.85† | 0.83±0.01 | 0.74±0.03 | 7.40±0.77* | 3.22±0.40† |
| *Renal section area (mm2)* | 53.9±3.7 | 34.1±4.3* | 186.5±22.9* | 124.1±18.0§ | 34.1±4.3 | 35.9±1.2 | 164.2±17.0* | 97.1±9.7† |
| *Cyst area (mm2)* | 9.3±3.6 | 6.3±2.9 | 117.7±18.2* | 66.8±17.0† | 5.7±1.3 | 5.1±1.9 | 108.4±11.3* | 51.8±6.8† |
| *Cyst area: renal section area (%)* | 17.8±7.8 | 15.5±5.5 | 63.1±5.8* | 53.4±6.5 | 16.3±2.1 | 14.3±5.7 | 66.0±3.0* | 53.4±4.0§ |
| *Serum glucose (mmol/L)* | 13.7±2.4 | 13.3±0.7 | 10.2±1.9 | 13.1±4.5 | 12.8±3 | 12.2±3.5 | 11.2±3.1 | 12.1±4 |
| *Serum albumin (g/L)* | 28±3 | 28±1 | 27±1 | 28±1 | 30±1 | 30±1 | 31±4 | 31±2 |
| **Week 16** | **n = 4** | **n = 4** | **n = 8** | **n = 10** | **n = 5** | **n = 4** | **n = 9** | **n = 6** |
| *Body weight (g)* | 309±25 | 316±19 | 242±48* | 301±29† | 187±18 | 185±6 | 184±31 | 184±16 |
| *Kidney weight (g)* | 2.2±0.1 | 2.0±0.2 | 17.2±3.0* | 13.5±2.6§ | 1.4±0.2 | 1.2±0.1 | 15.2±3.3* | 7.8±2.0† |
| *Kidney: body weight (%)* | 0.70±0.02 | 0.63±0.03 | 7.18±0.73* | 4.49±0.64† | 0.74±0.07 | 0.67±0.04 | 8.25±0.92* | 4.31±1.31† |
| *Renal section area (mm2)* | 53.9±3.7 | 34.1±4.3* | 186.5±22.9* | 124.1±18.0§ | 34.1±4.3 | 35.9±1.2 | 164.2±17.0* | 97.1±9.7† |
| *Cyst area (mm2)* | 12.8±5.2 | 11.0±3.7 | 153.3±24.7* | 119.5±22.3§ | 6.0±1.0 | 6.4±0.7 | 134.6±31.8* | 73.4±23.1§ |
| *Cyst area: renal section area (%)* | 21.5±7.4 | 19.0±5.6 | 67.8±4.3* | 63.9±4.1 | 16.2±2.2 | 17.0±1.6 | 68.6±4.8* | 58.4±9.0§ |
| *Serum glucose (mmol/L)* | 13.4±3.4 | 11.6±1.5 | 10.8±3.1 | 10.2±0.8 | 10.7±2.5 | 14.4±1.9 | 9.2±1.1 | 11.2±3.1 |
| *Serum albumin (g/L)* | 30±1 | 33±1 | 30±1 | 30±2 |  |  | 31±4 | 31±4 |

*p<0.001 versus age-matched NWI Lewis rat, †p<0.001 versus age-matched LPK NWI, ‡p<0.05 versus age-matched NWI Lewis rat, §p<0.05 versus age-matched NWI LPK rat
