# Supplementary material for: Increased water intake reduces long-term renal and cardiovascular disease progression in experimental polycystic kidney disease
Source: PLoS One. 2019 Jan 2;14(1):e0209186. doi: 10.1371/journal.pone.0209186 (PMC6314616; doi:10.1371/journal.pone.0209186)
Supplement: S2 Table — (DOCX) [file pone.0209186.s002.docx]

**S2 Table: Gender and effect of increased water intake on renal inflammation and myofibroblast infiltration at week 10**

|  | **Males** | | | | **Females** | | | |
| --- | --- | --- | --- | --- | --- | --- | --- | --- |
|  | **Lewis** | | **LPK** | | **Lewis** | | **LPK** | |
| *Variables* | **NWI** | **HWI** | **NWI** | **HWI** | **NWI** | **HWI** | **NWI** | **HWI** |
| **Week 10** | **n = 4** | **n = 4** | **n = 8** | **n = 8** | **n = 3** | **n = 4** | **n = 9** | **n = 8** |
| *Renal section area (mm2)* | 54.6±3.4 | 43.8±2.1‡ | 186.6±24.9* | 124.0±14.9§ | 34.2±3.0 | 35.9±1.2 | 131.2±17.7* | 97.1±10.2§ |
| *Myofibroblast infiltration (mm^2^)* | 1.0±0.7 | 0.9±0.4 | 10.3±6.1 | 7.7±3.9 | 1.5±1.6 | 1.4±0.5 | 9.3±5.2 | 5.5±2.7 |
| *Myofibroblast index (%)* | 2.0±1.3 | 2.3±1.2 | 10.5±5.3 | 10.7±4.5 | 4.5±4.0 | 4.3±1.3 | 13.9±8.2 | 10.1±4.7 |
| *Monocyte infiltration (mm^2^)* | 0.4±0.3 | 0.3±0.1 | 0.6±0.2 | 0.7±0.3 | 0.3±0.1 | 0.4±0.4 | 0.8±0.7 | 0.5±0.3 |
| *Monocyte index (%)* | 0.9±0.6 | 0.9±0.6 | 1.0±0.2 | 1.5±0.6 | 1.0±0.3 | 1.3±1.1 | 1.3±1.0 | 1.2±0.6 |
| *Interstitial collagen deposition (mm^2^)* | 4.9±1.7 | 1.1±0.6 | 33.1±23.6 | 19.5±8.4 | 1.9±0.7 | 2.1±1.7 | 22.4±9.3 | 14.6±5.8 |
| *Interstitial collagen deposition index (%)* | 9.0±0.3 | 2.5±1.3 | 19.0±15.9 | 15.9±7.2 | 5.7±2.1 | 6.0±4.7 | 13.8±4.7 | 15.0±5.1 |
| **Week 16** | **n = 4** | **n = 4** | **n = 8** | **n = 8** | **n = 3** | **n = 4** | **n = 9** | **n = 8** |
| *Renal section area (mm2)* | 56.9±6.3 | 55.6±3.4‡ | 230.7±31.9* | 179.6±26.3§ | 37.2±4.7 | 37.5±3.2 | 200.2±45.5* | 120.6±28.4§ |
| *Myofibroblast infiltration (mm^2^)* | 1.5±0.9 | 1.4±0.5 | 11.6±4.5‡ | 13.5±2.7 | 1.1±0.3 | 0.7±0.5 | 10.8±8.3‡ | 4.8±2.8 |
| *Myofibroblast index (%)* | 2.9±1.8 | 2.6±0.9 | 11.4±5.8 | 15.6±5.4 | 3.1±0.8 | 2.3±1.8 | 13.2±9.0 | 7.7±3.9 |
| *Monocyte infiltration (mm^2^)* | 0.4±0.2 | 0.3±0.2 | 0.6±0.2 | 0.4±0.1 | 0.2±0.1 | 0.4±0.6 | 0.5±0.3 | 0.5±0.1 |
| *Monocyte index (%)* | 0.9±0.6 | 0.7±0.6 | 0.8±0.3 | 0.7±0.2 | 0.5±0.4 | 1.1±1.5 | 0.9±0.7 | 1.1±0.3 |
| *Interstitial collagen deposition (mm^2^)* | 4.5±3.5 | 3.9±1.6 | 43.7±21.4 | 27.0±11.8 | 3.4±1.7 | 1.7±1.9 | 58.5±42.2 | 21.8±8.5§ |
| *Interstitial collagen deposition index (%)* | 8.3±6.7 | 7.3±2.9 | 20.1±12.8 | 15.2±7.4 | 9.2±4.0 | 4.5±5.0 | 29.3±20.7 | 19.2±9.1 |

*p<0.001 versus age-matched NWI Lewis rat, †p<0.001 versus age-matched LPK NWI, ‡p<0.05 versus age-matched NWI Lewis rat, §p<0.05 versus age-matched NWI LPK rat
