# Supplementary material for: Increased water intake reduces long-term renal and cardiovascular disease progression in experimental polycystic kidney disease
Source: PLoS One. 2019 Jan 2;14(1):e0209186. doi: 10.1371/journal.pone.0209186 (PMC6314616; doi:10.1371/journal.pone.0209186)
Supplement: S3 Table — (DOCX) [file pone.0209186.s003.docx]

**S3 Table: Gender and effect of increased water intake on renal function, serum sodium and albumin.**

|  | **Males** | | | | **Females** | | | |
| --- | --- | --- | --- | --- | --- | --- | --- | --- |
|  | **Lewis** | | **LPK** | | **Lewis** | | **LPK** | |
| *Variables* | **NWI** | **HWI** | **NWI** | **HWI** | **NWI** | **HWI** | **NWI** | **HWI** |
| **Week 10** | **n = 4** | **n = 4** | **n = 8** | **n = 8** | **n = 3** | **n = 4** | **n = 9** | **n = 8** |
| *Serum creatinine (umol/L)* | 24±7 | 23±3 | 53±32 | 50±17 | 28±6 | 28±3 | 40±4‡ | 48±8 |
| *Serum urea (mmol/L)* | 6.8±2 | 6.1±0.6 | 21.6±7.1* | 9.2±3.4† | 6±0.2 | 5.2±0.8 | 19.1±4.5* | 9.6±2.6† |
| *Urine PCR (mg/mmol Cr)* | 51.6±42.4 | 82.9±62.0 | 694.9±851.4 | 362.0±147.2 | 31.4 | 82.8+32.8 | 139.9±69.7 | 46.3±16.6§ |
| *CrCl/BW(ml/min/g)* | 7.00±2 | 8.50±1 | 3.00±1.07* | 3.50±1.31 | 5.67±2.08 | 6.25±1.50 | 2.67±0.71‡ | 2.50±0.53 |
| *Serum sodium (mmol/L^)^* | 143±1 | 142±5 | 146±5 | 129±11 | 146±1 | 142±2 | 148±2 | 145±3 |
| **Week 16** | **n = 4** | **n = 4** | **n = 8** | **n = 8** | **n = 3** | **n = 4** | **n = 9** | **n = 8** |
| *Serum creatinine (umol/L)* | 35±13 | 27±2 | 114±49‡ | 78±36 | 31±9 | 39±8 | 73±22‡ | 58±17 |
| *Serum urea (mmol/L)* | 8.1±1.8 | 6.4±0.5 | 31.6±10.4* | 19±8.1† | 6.5±0.8 | 5.1±0.6 | 26.7±7.2* | 12.4±3.5† |
| *Urine PCR (mg/mmol Cr)* | 25.5±17.8 | 179.6±138.3 | 1716.1±1854.6 | 272.6±178.0§ | 15.6 | 119.2±47.7 | 686.8±530.6 | 470±570.4 |
| *CrCl/BW(ml/min/g)* | 5.25±1.71 | 6.5±1.29 | 1.38±1.41* | 2.33±1.32 | 6±1.22 | 4.25±0.95 | 1.89±1.36* | 1.4±1.51 |
| *Serum sodium (mmol/L^)^* | 143±5 | 144±3 | 144±3 | 142±2 | 147±9 | 148±4 | 147±6 | 138±14 |

Urine PCR; urine protein creatinine ratio; CrCl/BW, creatinine clearance corrected for body weight. Values represented as mean ± SD.

*p<0.001 versus age-matched NWI Lewis rat, †p<0.001 versus age-matched LPK NWI, ‡p<0.05 versus age-matched NWI Lewis rat, §p<0.05 versus age-matched NWI LPK rat
