# Supplementary figures and images for: Increased water intake reduces long-term renal and cardiovascular disease progression in experimental polycystic kidney disease
Source: PLoS One. 2019 Jan 2;14(1):e0209186. doi: 10.1371/journal.pone.0209186 (PMC6314616; doi:10.1371/journal.pone.0209186)

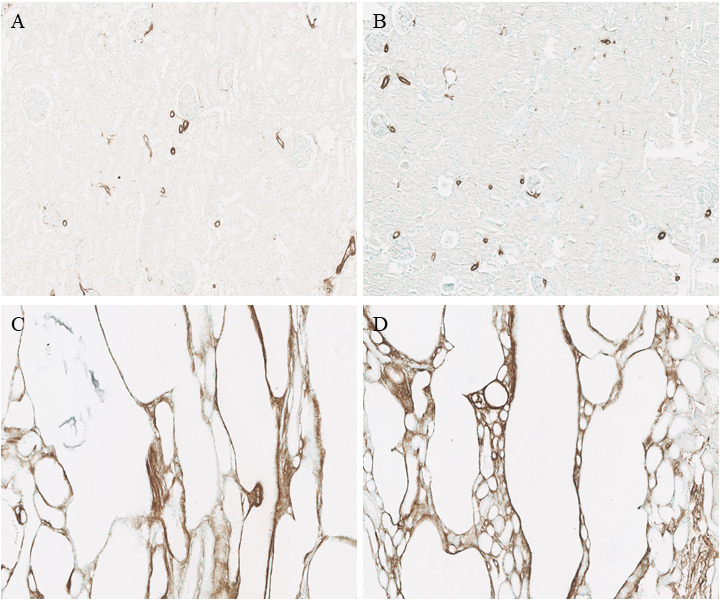

Supplement: S1 Fig — (A) Lewis NWI, (B) Lewis HWI, (C) LPK NWI, (D) Lewis HWI. Please see methods section for further details on staining protocol. (TIF) [file pone.0209186.s004.tif]

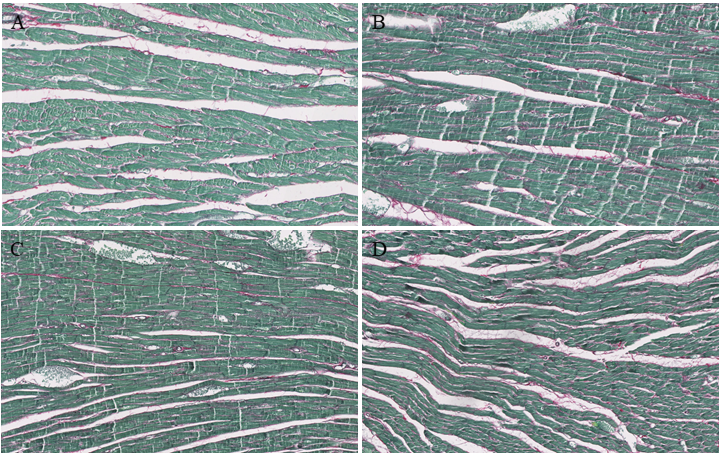

Supplement: S2 Fig — (A) Lewis NWI, (B) Lewis HWI, (C) LPK NWI, (D) Lewis HWI. Red-stained areas are consistent with the deposition of collagen. Please see methods section for further details on staining protocol. (TIF) [file pone.0209186.s005.tif]
